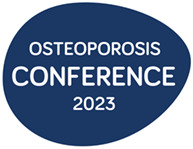# Abstracts from the Osteoporosis 2023 Conference, Royal Osteoporosis Society, September 13–14, University of Manchester, UK

**DOI:** 10.1002/jbm4.10815

**Published:** 2023-09-14

## Abstract